# Structural Bioinformatics and Protein Docking Analysis of the Molecular Chaperone-Kinase Interactions: Towards Allosteric Inhibition of Protein Kinases by Targeting the Hsp90-Cdc37 Chaperone Machinery

**DOI:** 10.3390/ph6111407

**Published:** 2013-11-11

**Authors:** Nathan Lawless, Kristin Blacklock, Elizabeth Berrigan, Gennady Verkhivker

**Affiliations:** 1 School of Computational Sciences and Crean School of Health and Life Sciences, Schmid College of Science and Technology, Chapman University, One University Drive, Orange, CA 92866, USA; E-Mails: lawle108@mail.chapman.edu (N.L.); black127@mail.chapman.edu (K.B.); berri104@mail.chapman.edu (E.B.); 2 Department of Pharmacology, University of California San Diego, 9500 Gilman Drive, La Jolla, CA 92093, USA

**Keywords:** molecular chaperones, protein kinases, protein docking, protein-protein interactions, allosteric binding sites, drug discovery

## Abstract

A fundamental role of the Hsp90-Cdc37 chaperone system in mediating maturation of protein kinase clients and supporting kinase functional activity is essential for the integrity and viability of signaling pathways involved in cell cycle control and organism development. Despite significant advances in understanding structure and function of molecular chaperones, the molecular mechanisms and guiding principles of kinase recruitment to the chaperone system are lacking quantitative characterization. Structural and thermodynamic characterization of Hsp90-Cdc37 binding with protein kinase clients by modern experimental techniques is highly challenging, owing to a transient nature of chaperone-mediated interactions. In this work, we used experimentally-guided protein docking to probe the allosteric nature of the Hsp90-Cdc37 binding with the cyclin-dependent kinase 4 (Cdk4) kinase clients. The results of docking simulations suggest that the kinase recognition and recruitment to the chaperone system may be primarily determined by Cdc37 targeting of the N-terminal kinase lobe. The interactions of Hsp90 with the C-terminal kinase lobe may provide additional “molecular brakes” that can lock (or unlock) kinase from the system during client loading (release) stages. The results of this study support a central role of the Cdc37 chaperone in recognition and recruitment of the kinase clients. Structural analysis may have useful implications in developing strategies for allosteric inhibition of protein kinases by targeting the Hsp90-Cdc37 chaperone machinery.

## 1. Introduction

**Figure 1:**
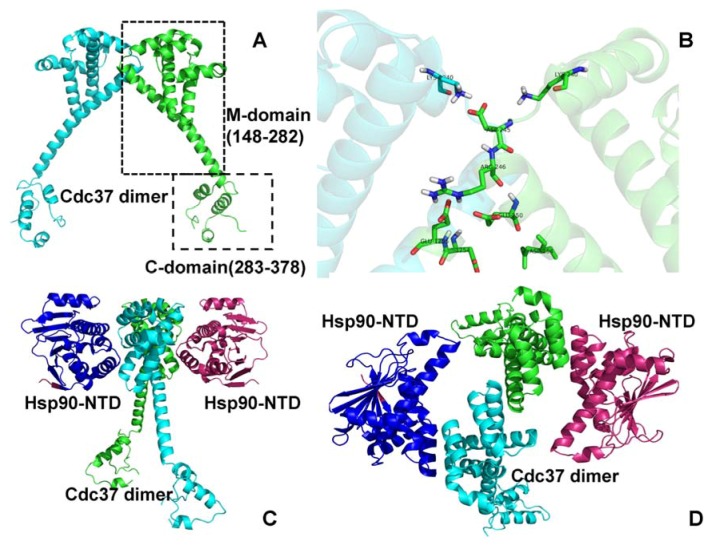
Structural Characterization of the Cdc37 Chaperone. (**A**) The crystal structure of human Cdc37 dimer from the complex with the NTD of yeast Hsp90 (pdb id 1US7) [50]. The Cdc37 monomers are colored in cyan and green. The middle domain (residues 148–282) is involved in recognition of protein kinase clients. The C-terminal domain (residues 283–378) is involved in dimerization. (**B**) A close-up of the intermonomer interface from the crystal structure of the Cdc37 dimer [50]. The front view (**C**) and the top view (**D**) of the Cdc37 dimer (colored in green and cyan) bound to the NTDs of yeast Hsp90 (colored in blue and pink). The crystal structure of human Cdc37 dimer (pdb id 1US7) [50], the crystal structure of the isolated Cdc37 M-domain (pdb id 2W0G) [52] and the NMR structure of the complex of the human Cdc37 M-domain with the N-terminal domain of human Hsp90 (pdb id 2K5B) [52] have provided structural information used in this study.

Molecular chaperones are essential proteins that have evolved to assist and facilitate conformational development, folding and stability of a diverse repertoire of protein clients inside the cell, including a wide range of protein kinases [1,2,3,4,5,6,7,8,9,10]. The human protein kinome presents one of the largest protein families that orchestrate functional processes in complex cellular networks during growth, development and stress response [11,12,13]. Allosteric regulation and support of protein kinase activity by molecular chaperones underlie the fundamental role of chaperones in protein synthesis, refolding and degradation [8,9,10]. The molecular chaperones Hsp70 and Hsp90 act cooperatively in the biogenesis of protein kinases by assisting in the initial folding of the polypeptide chain and protecting nearly-folded, activation-competent protein states from degradation and aggregation. Cell division cycle protein 37 (Cdc37) is a highly specialized co-chaperone that independently and in coordination with Hsp90 can facilitate protein folding and maintain stabilization of protein kinases during maturation until they attain their full biological activity [14,15,16]. A fundamental role of the Hsp90-Cdc37 chaperone tandem as a coordinated “house-keeping crew” that mediates maturation of protein kinase clients and supports kinase functional activity appeared to be essential for the integrity and viability of signaling pathways involved in cell cycle control and organism development [17,18]. Although both molecular chaperones are needed to develop and stabilize active kinase forms capable of executing signaling processes in the cell, Cdc37 lacking the Hsp90-binding domain can still bind various kinases [14]. A series of early biochemical studies has unveiled the essential role of Cdc37 in maintaining oncogenic kinase clients and modulating nucleotide-dependent conformational changes of Hsp90 [19,20,21,22,23,24,25,26]. Recent findings have revealed a direct involvement of Cdc37 in biogenesis of the protein kinome and quality control by protecting nascent polypeptide chains from degradation [27,28]. Genomic and proteomic studies in yeast have shown that, whereas a relatively small population of the cellular kinome requires Hsp90, many more kinases depend on Cdc37 [29]. Several studies have provided a convincing evidence of an Hsp90-independent chaperone function of Cdc37 in supporting kinase activity, raising the question whether Cdc37 is a co-chaperone of Hsp90 or Cdc37 may employ Hsp90 in a supporting role [14,27,28,29]. Although it is not yet clear how Hsp90 is involved in maturation of Cdc37-interacting kinases, these studies have confirmed an important role of Cdc37 as an adaptor which may “decide” if the recruitment of a kinase to the chaperone system is warranted for activation. The Hsp90-Cdc37 chaperone system is required to maintain activity and stability of many tumor-inducing signaling protein kinases. As a result, inhibition of the chaperone machinery may lead to a combinatorial disruption of numerous oncogenic pathways while simultaneously achieving tumor cell specificity [30,31,32]. The crystal structures of Hsp90 from yeast [33,34], *E. coli* HtpG [35], and Grp94 homologue [36] have revealed a homodimer that operates in a functional cycle associated with the ATP binding and hydrolysis. Structural and functional versatility of the molecular chaperone is provided by a modular architecture with three well-defined domains: an N-terminal domain (NTD) responsible for ATP binding, a Middle domain (M-domain), which completes the ATPase site and binds client proteins, and a C-terminal domain (CTD) that is required for dimerization [37,38]. Structural and functional studies [39,40,41,42,43,44] have suggested a stochastic mechanism of the Hsp90 ATPase cycle, according to which the inherent conformational flexibility of the molecular chaperone allows for functional adaptation to binding with co-chaperones and protein clients. The human Cdc37 protein structure can be divided into three domains where the N-terminal domain (residues 1–147) and the middle domain (residues 148–282) recognize protein kinase clients and Hsp90, while the C-terminal domain (residues 283–378) is primarily involved in dimerization (Figure 1) [45]. The phosphorylated form of the N-terminal domain of Cdc37 was implicated in mediating kinase stabilization and maturation [46,47,48]. The middle domain is the most stable region of Cdc37, which is resistant to proteolytic digestion and contains both Hsp90 and kinase recognition sites [49]. The crystal structure of the human Cdc37 construct (residues 148–348) in the complex with the yeast Hsp90-NTD has revealed a Cdc37 dimer bound to the “lid” segment of the Hsp90-NTD and intruding into the Hsp90 nucleotide binding pocket [50]. These interactions formed between the middle domain of Cdc37 and the Hsp90-NTD can inhibit the ATPase activity of Hsp90 by preventing dimerization and disrupting the Hsp90 ATPase cycle [50,51]. A solution state NMR study of the complex between the middle domain of human Cdc37 (residues 148–276) and human Hsp90-NTD has produced a monomeric structure of Cdc37 forming a compact hydrophobic binding interface with the Hsp90-NTD [52]. These structural studies have suggested that multiple factors can be implicated in the mechanism of Cdc37-mediated inhibition of the ATPase activity: (a) the hydrogen bonding between Cdc37-R167 and catalytic residue Hsp90-E33 can prevent hydrolysis of ATP, although it could still allow for ATP binding; (b) the interactions of Cdc37 with the Hsp90 lid can interfere with the formation of the closed lid conformation and trigger arrest of the Hsp90-ATPase cycle in the open Hsp90 conformation, thus blocking access of the catalytic residues to the nucleotide site required for ATP hydrolysis. Structural studies of the Hsp90 and Cdc37 chaperones have culminated in the electron microscopy (EM) reconstruction of the Hsp90-Cdc37-kinase complex [53]. The asymmetric assembly of an Hsp90 dimer bound to a Cdc37 monomer and cyclin-dependent kinase 4 (Cdk4) has revealed that the NTD of one Hsp90 monomer remains in a catalytically competent conformation whereas the other Hsp90-NTD is hinged away from a dimerization arrangement and bound to Cdc37.

According to this pioneering study, conformational changes of the Hsp90-Cdc37 chaperone during ATPase cycle are coupled to kinase activation via a complex mode of interactions: the N-terminal lobe of Cdk4 associates with the Cdc37 monomer and the Hsp90-NTD, while the C-terminal kinase lobe binds to the middle domain of Hsp90 [53]. Structural and biochemical experiments have indicated that Cdc37 would bind to a semi-open form of Hsp90 that was observed in the crystal structure of the ADP-bound Hsp90 dimer [49,50,51]. SAXS studies have suggested a model in which a Cdc37 dimer would bind to both N-terminal domains of the Hsp90 dimer, causing a contraction of the apo-Hsp90 and preventing closure of the nucleotide site needed for the ATPase hydrolysis [49]. According to this mechanism, a Cdc37-mediated arrest of the Hsp90-ATPase cycle in a specific functional form of Hsp90 would allow for a dynamic loading and release of kinase clients to the Hsp90 machinery [49,50,51]. Two-hybrid analysis of the yeast Hsp90 [54] and biochemical *in vivo* studies of Hsp90-cochaperone binding [55] have confirmed these assertions by demonstrating that Hsp90 progression between the ADP-bound and the ATP-bound states can be blocked by mutations of the Hsp90-NTD residues implicated in Cdc37 binding. These experiments corroborated with the results of allosteric inhibition of the Hsp90-Cdc37 interactions [56,57,58] confirming structural preferences of Hsp90 for a nucleotide-free state in the complex with Cdc37 [56].

The initial biochemical *in vitro* assays have identified that a conserved GXGXXG motif of the glycine loop in the N-terminal kinase lobe may be required for Cdc37 recognition [59]. Mutagenesis experiments have revealed that G15A and G18A variants within this motif were critical for the Cdc37-Cdk4 complex formation [59]. Subsequent studies have shown that a 20-residue region (residues 181–200) of the middle domain of Cdc37 is sufficient and a five-residue motif (residues 190-LVIWCI-195) may be essential for Cdc37 binding with protein kinase clients [60]. Although this region resides in a close proximity of the Hsp90-Cdc37 interface, a cooperative nature of chaperone-client interactions would manifest in binding of the middle Cdc37 domain with both Hsp90-NTD and the N-terminal kinase lobe [60]. Phage display and liquid chromatography-tandem mass spectrometry experiments have later identified a truncated GXFG motif from the canonical glycine-rich loop (GXGXXG) of protein kinases as an important Cdc37-interacting region [61]. Although the N-terminally truncated form of Cdc37 (residues 181–378) may be sufficient to preserve kinase binding activity of Cdc37, it could also allow for recognition of both client and non-client protein kinases [60,61]. Functional dissection of protein kinase motifs has demonstrated that the initial recruitment and binding with the Hsp90-Cdc37-chaperone involves a cooperative effort of multiple segments from the N-terminal and C-terminal lobes of the catalytic domain [62,63,64]. The important molecular determinants of Cdc37-kinase recognition may be localized near the αC-helix and the adjacent αC-β4 loop motif in the N-terminal lobe of the catalytic kinase domain, while the region connecting the N-terminal and the C-terminal kinase lobes may be required for minimal recognition by Hsp90 [62,63,64]. Although sequence variations in this region could not differentiate between client and nonclient kinases, mutations in the αC-β4 loop had a strong effect on chaperone binding [65,66]. As a result of these findings, it was contemplated that the chaperone system could distinguish kinase clients by recognizing conformational instability of the N-terminal lobe and the αC-β4 loop motif. However, the existing experimental data could not quantify this mechanism or unambiguously attribute the primary recognition event to either Hsp90 or Cdc37 components of the chaperone machinery [67,68].

A number of proteomic approaches have been undertaken to explore the Hsp90 regulated proteome including global proteome profiling [69,70,71] and cell-based interrogation exploiting proteomic response to immobilized Hsp90 inhibitors [72,73,74]. These studies have identified two major global effects of Hsp90 inhibition on the cellular proteome which are the increase of the unfolded protein response and the decrease of kinase associated processes. A high-throughput study of Hsp90 interactions using luminescence-based mammalian interactome mapping has provided a first quantitative assessment of client kinase binding [71]. The observed correlation between Hsp90-kinase and Cdc37-kinase interactions has demonstrated that both chaperones act concertedly to support functional activity of client kinases during maturation. We have recently reported a series of computational investigations of the Hsp90 chaperone [75,76,77,78] and oncogenic protein kinases [79,80] that dissected the dynamics and allosteric interactions of these proteins with an atomic level analysis of the conformational motions and the inter-domain communication pathways. In this work, we used structural bioinformatics analysis of the interacting sites and protein docking to probe the allosteric nature of the Hsp90-Cdc37 binding with Cdk4 kinase client. The results of docking simulations suggest that the kinase recognition and recruitment to the chaperone system may be primarily determined by Cdc37 targeting of the N-terminal kinase lobe.

## 2. Results and Discussion

### 2.1. Bioinformatics Analysis of the Interaction Maps

**Figure 2:**
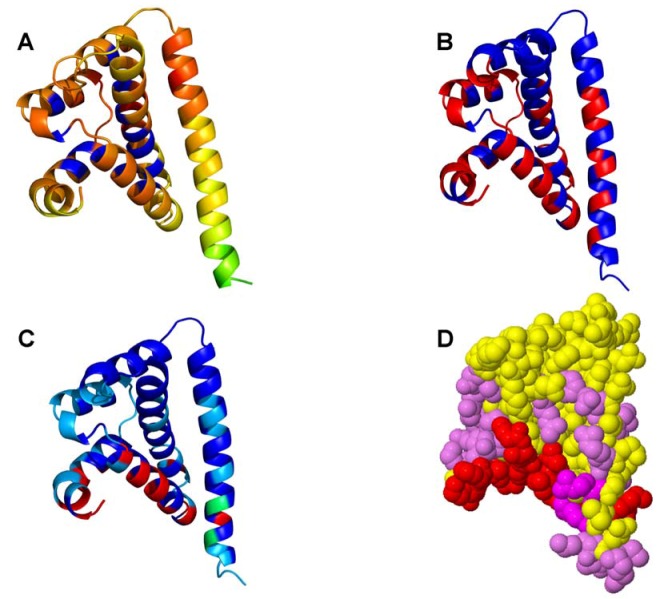
The Interaction Maps of the Cdc37 Chaperone. The crystal structure of the isolated Cdc37 M-domain (pdb id 2W0G) [52] was used for mapping interaction preferences of Cdc37. The protein-protein interaction predictor methods ProMate (**A**), InterProSurf (**B**) and SPPIDER (**C**) was used to build a consensus scoring of probable interfacial residues shown in spheres (**D**). The distribution of interfacial regions in the crystal structures is represented by a color scheme, where blue corresponds to the regions with the lowest probability and red corresponds to the regions with the highest probability to interface with binding partners. The kinase-recognition region (residues 190-LVIWCI-195) received the highest scores (colored in red spheres in **D**).

The results of biochemical studies [60,61,62,63] suggested that Cdc37 provides initial recognition of the kinase family by probing the N-terminal kinase lobe and forming short-lived encounter complexes. These transient interactions, however, are likely to be prolonged and enhanced for client kinases, thereby promoting stabilization and chaperone-assisted activation of the kinase. On the other hand, proteomics studies [71] have proposed a model of combinatorial-based initial recognition of the kinase folds that is followed by thermodynamics-based screening of clients and nonclients. To elucidate further the mechanisms of kinase recognition and recruitment to the chaperone, we analyzed general interaction propensities of the notable kinase clients (Figure 2). According to our objective, structural analysis and comparison of dynamic and interaction preferences of the kinase clients may help to clarify whether the αC-β4/αC-helix motif has a universal functional role that could be tailored for general recognition of kinase folds and specific regulatory functions. A panel of various protein-protein interaction predictor methods including ProMate [81], WHISCY [82], Consurf [83], InterProSurf [84], PPI-Pred [85], and SPPIDER [86] was used to build a consensus scoring of probable interfacial residues [87] for the kinase clients and the Cdc37 chaperone. The crystal structure of the human Cdc37 C-domain [50], the crystal structure of the M-domain of Cdc37, and the NMR structure of the human Cdc37 in the complex with the human Hsp90-NTD [52] have provided the source of experimental information for probing the interfacial preferences of Cdc37. We focused our analysis on the middle domain of Cdc37 involved in binding with Hsp90 and kinases [49].

In agreement with the experimental results [60,61], we identified a helical motif of Cdc37 (residues 187-ANYLVIWCID-196), that was implicated in recognition of kinase clients, as the most probable interfacial region (colored in red) of the co-chaperone. The core of this region (residues 190-LVIWCI-195) that received the highest scores was found to be essential in the biochemical studies of chaperone-kinase interactions [60,61]. Interestingly, the interaction maps assigned only intermediate interfacial scores to the M-domain residues [52]. Accordingly, the principal interfacial region of Cdc37 is responsible for recognition of kinase clients, in line with a central role of Cdc37 during kinase recruitment to the chaperone system. 

**Figure 3:**
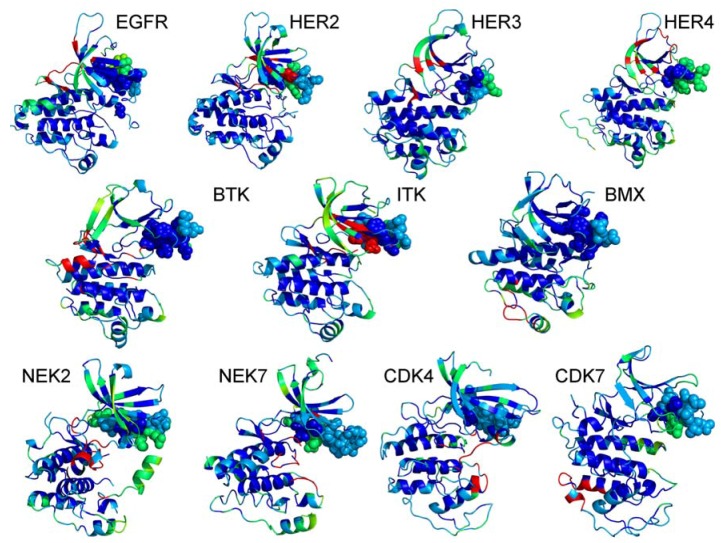
The Interaction Maps of the Protein Kinase Clients. Structural mapping of interaction preferences of various kinase clients onto the crystal structures. The protein-protein interaction predictor methods ProMate , InterProSurf and SPPIDER (C) was used to build a consensus scoring of probable interfacial residues. The distribution of interfacial regions in the crystal structures is represented by a color scheme, where blue corresponds to the regions with the lowest probability and red corresponds to the regions with the highest probability to interface with binding partners.

The interfacial regions of various kinase clients are mainly distributed on the exposed N-terminal lobe, and involve the P-loop, β1, β2, and β3-strands (Figure 3). At the same time, structural core of the catalytic domain is largely insulated from exposure to the binding partners. The αC-β4/αC-helix motif is located between these kinase compartments and is often a mixed bag of residues with different propensity levels for protein interactions (refer to a sphere-based representation in Figure 3). The inspection of the interaction maps may indicate that kinase recognition by Cdc37 may initially proceed by probing the exposed motifs in the N-terminal lobe. This event of “kinase fold registration” may be accompanied by the formation of short-lived, non-specific intermediate complexes. In a subsequent step, Cdc37 would “diagnose” a kinase partner as its client (or nonclient) and begin recruitment to the chaperone system by recognizing structural and dynamical preferences of the αC-β4/αC-helix motif.

This hierarchical model of chaperone-kinase recognition may explain and reconcile some conflicting observations from different biochemical studies. The N-terminal kinase lobe and the P-loop residues were recognized in early biochemical studies as important contributors of the Cdc37-kinase interface [61]. However, later developments have shown that mutations within the P-loop (GXGXYG) may not fully disrupt the regulatory interactions and single-handedly abort binding of kinase clients to Cdc37 [88]. According to our model, these observations may not be mutually exclusive and echo the notion that the αC-helix, rather than the P-loop, is likely the principal determinant of kinase binding with the chaperone. The dynamic and interaction maps of the kinase clients have yielded a plausible mechanistic model of kinase-chaperone binding as a successive cascade of recognition events. The initial recognition of the kinase fold by Cdc37 may be primarily determined by the interactions with the exposed P-loop of the N-terminal lobe. Subsequent steps of recognizing kinases with a low catalytic activity and assessing their functional “eligibility” for chaperone assistance would be determined by the interactions with the αC-helix, a region that is critical for both allosteric kinase activation and chaperone recruitment.

### 2.2. Experimentally-Guided Docking of the Hsp90-Cdc37-Cdk4 Interactions

The biochemical characterizations of chaperone interactions with protein kinase clients [60,61,62,63,64,65,66,67,68] in combination with global mapping of the Hsp90 regulated proteome [71,72,73,74] have provided a significant source of experimental information that can be employed in structural modeling of Hsp90-Cdc37-kinase interactions. Computational docking strategies often integrate diverse sets of experimental data such as mutagenesis data and NMR chemical shift perturbations [89] as experimental restraints to guide sampling of protein-protein complexes. The EM reconstruction of the Hsp90-Cdc37-Cdk4 complex has offered unparalleled insight into the stoichiometry, topology and shape of this multi-protein assembly [53], yet the details of the protein-protein interfaces remain largely unknown. Equipped with the extensive collection of structure-functional data about chaperone-kinase client interactions, we attempted to map at the atomic level the intermolecular interfaces of the chaperone complex with the Cdk4 client.

**Figure 4:**
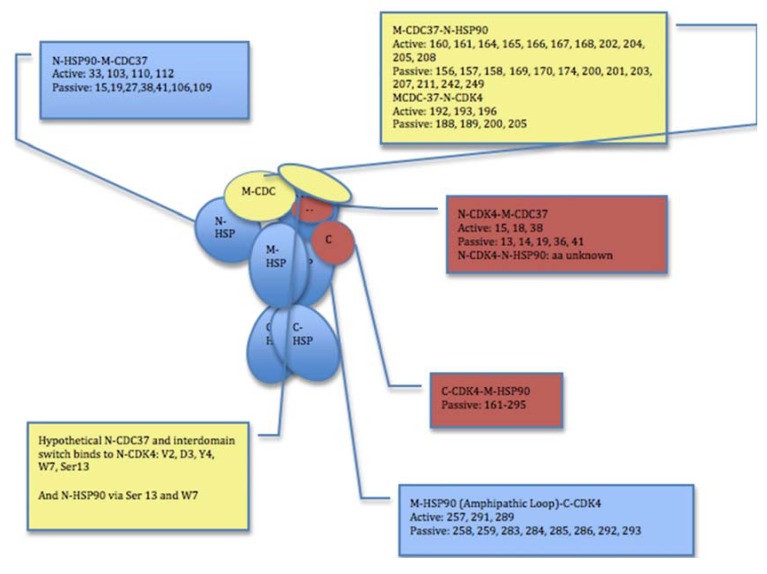
A Schematic Map of Experimentally-Guided Interaction in HADDOCK Simulations. The cartoon outlines and summarizes the selections of active/passive residues and experimental restraints for HADDOCK simulations. Definition of active residues was derived driven from EM reconstruction data of the Hsp90-Cdc37-Cdk4 [53] and mutagenesis studies of chaperones and kinase clients [60,61,62,63,64,65,66,67,68,69,70,71,72,73,74]. A combination of experimental restraints and bioinformatics-based analysis of binding interfaces was used to determine sets of active and passive residues for HADDOCK.

Our approach is based on data-driven HADDOCK methodology [90,91,92] that integrates mutagenesis data and NMR chemical shift perturbations as experimental restraints in docking of protein complexes. In this approach, the amino acids of interacting partners are designated as “active” and “passive” residues reflecting their role in binding. The residues are designated as active if they were experimentally detected to contribute to the intermolecular interface. Passive residues are defined as those within a 5Å radius to the active residues. Structural and functional information is then converted into a series of Ambiguous Interaction Restraints (AIR) used by HADDOCK. The selection of active residues forming the AIR templates (Figure 4) is generally based on two primary sources of experimental information: structural topology of the Hsp90-Cdc37-Cdk4 complex obtained from the EM study [53], and the functional significance of specific residues that is inferred from biochemical and structural studies of Hsp90 and kinase clients [60,61,62,63,64,65,66,67,68,69,70,71,72,73,74]. The AIR templates are modeled using an ambiguous distance restraint between all atoms of the source residue to all atoms of the target residue that are assumed to be in the interface [90,91,92].

The assembled sets of active and passive residues forming the AIR templates (Figure 4) reflect the general topology of the Hsp90-Cdc37-Cdk4 assembly, namely the fact that (a) the M-domain of Cdc37 interacts with the Hsp90-NTD; (b) the N-terminal lobe of Cdk4 associates with the Cdc37 monomer and the Hsp90-NTD, and (c) the C-terminal kinase lobe binds to the middle domain of Hsp90 [53]. Consistent with the NMR studies of Cdc37-Hsp90 interactions [52], we selected Lys-160, His-161, Met-164, Leu-165, Arg-166, Arg-167, Trp-168, Trp-193, Lys-202, Ala-204, and Leu-205 of the M-domain of Cdc37 as active residues. The structural core of the Cdc37-Hsp90 interface involves the Hsp90-NTD residues (Ala-117, Ala-121, Ala-124, Ala-126, Met-130, and Phe-134) as determined in the complex of the human Cdc37 M-domain with the N-terminal domain of human Hsp90 (pdb id 2K5B) [50,52]. According to biochemical studies [49], Cdc37 would bind to Hsp90 in the ADP-bound, nucleotide-free state. As result, the experimental restraints and active residue selection obtained from human Hsp90 [52,53] were mapped onto the ADP-bound crystal structure of the HtpG structure [35,42] using a homology comparison. The residues 190-LVIWCID-196 from a kinase-interacting helix of Cdc37 (184-EETANYLVIWCIDLEVE-200) [60,61] were designated as active residues and defined the Cdc37-Cdk4 binding interface.

A set of experimental restraints defining the active kinase residues is based on biochemical evidence that Gly-13, Gly-15, Gly-18, and Lys-35 of the glycine-rich loop as well as the β4 and β5 strands of the N-terminal lobe are involved in binding with Cdc37 [59] (Figure 4). Bioinformatics-based analysis and annotation of Hsp90-Cdc37 interactions with kinase clients have strongly indicated that the αC-β4 loop and the αC-helix motifs of the N-terminal lobe are central determinants of Cdk4-Cdc37 recognition. As a result, in another set of experimental restraints, the α-C-helix residues 50-PISTVREVALLRRLAEFE-67 and the adjacent αC-β4 loop motif (68-HPNVVRL-74) (pdb id 2W9Z) [93] are designated as active residues of Cdk4.

The docking protocol consists of three consecutive stages: (a) randomization of orientations followed by rigid body energy minimization (EM); (b) semi-flexible simulated annealing in torsion angle space (TAD-SA), which consists of a rigid body MD search and first round of simulated annealing, followed by a second round semi-flexible simulated annealing during which side chains at the interface are free to move. A third round of semi-flexible simulated annealing is the next step of the search during which both side chains and backbone at the interface are free to move. A final refinement in Cartesian space with explicit solvent concludes the run [92]. A large number of independent HADDOCK runs (>1000) were initiated using the crystal structures of Cdc37, Cdk4 and the conformational ensemble of ADP-bound derived Hsp90 conformations. In the initial rigid body docking phase, 2000 rigid-body structures were generated. During first round of rigid body search and simulated annealing docked solutions were judged by the HADDOCK score with standard weights of the individual contributions as defined in [90,91,92]:

*HADDOCK Score* = *E_vdw_* + *E_elec_* + *E_AIR_* (1)

In this equation, *E_vdw_* is the van der Waals energy, *E_elec_* is the electrostatic energy, and *E_AIR_* is the distance restraint contribution of AIRs. The best 200 docked models were submitted to cycles of the semi-flexible simulated annealing and final water refinement. After the water refinement stage the HADDOCK score was calculated as the following weighted sum:

*HADDOCK Score* = 1.0*E_vdw_* + 0.2*E_elec_* + 0.1*E_dist_* + 1.0*E_solv_* (2)

where *E_solv_* the solvation energy is term, and *E_dist_* is the distance restraints energy contribution that includes both unambiguous interaction restraints and AIRs.

The nonbonded intermolecular interactions were calculated with an 8.5 Å cutoff using the OPLS parameters. The dielectric constant epsilon was set to 10 in the vacuum part of the protocol and to 1 for the explicit solvent refinement. The secondary structure elements were kept intact during the simulated annealing refinement through hydrogen bond and dihedral angle restraints. HADDOCK tools were used for clustering and scoring of docking solutions. The cutoff distance of 10 Å and a minimum cluster size of five structures were used in clustering of docked poses. The final selection of clusters was based on the HADDOCK score and the best scored structures from each cluster were reported and analyzed.

**Figure 5:**
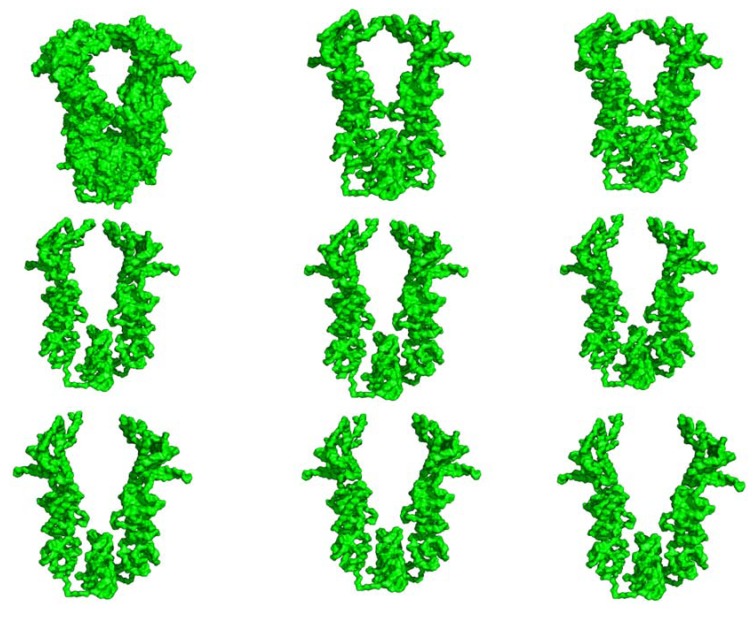
Conformational Ensemble of the Hsp90 Chaperone Employed in Docking Simulations.

**Figure 6:**
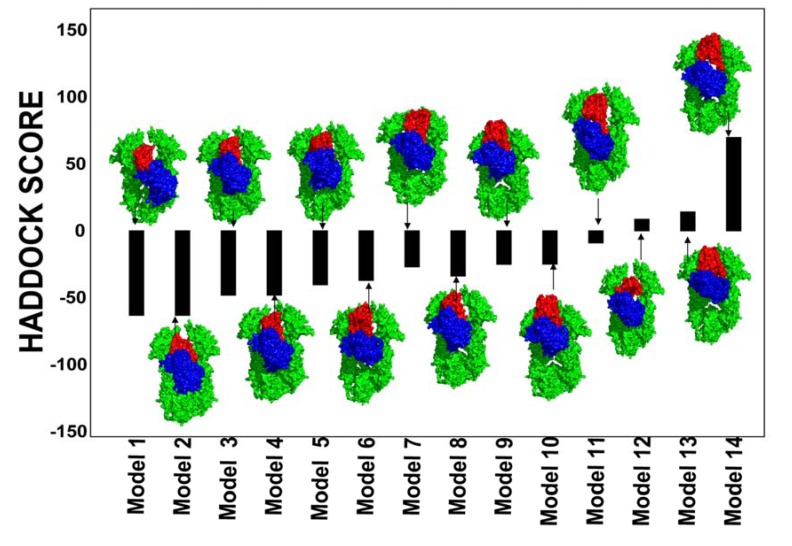
Structures and Energies of the Hsp90-Cdc37-Cdk4 Docked Complexes. The presented docked solutions are the low-energy binding modes obtained after stages of 3-body docking, refinement and clustering. Structures of the Hsp90-Cdc37-Cdk4 complexes are aligned with their respective HADDOCK score. A surface-based protein representation is used. The Hsp90 dimer is shown in green, Cdc37 is colored in red and Cdk4 is displayed in blue. Structural analysis of the docked solutions shows a gradual improvement and consolidation of the Hsp90-Cdc37 and Cdc37-Cdk4 intermolecular interfaces as the HADDOCK score of the docked complexes improved.

Docking simulations incorporated the notion that Hsp90-Cdc37 binding would likely favor an ADP-bound form of Hsp90 [49]. To emulate a dynamic nature of Hsp90-Cdc37-Cdk4 assembly, we generated a conformational ensemble of the ADP-bound HtpG form by using the DC-ENM server [94] (Figure 5). This approach utilizes the lowest normal modes obtained from the GNM analysis of the HtpG crystal structure and distance constraints obtained the experimental data. To obtain an adequate initial representative of the chaperone ensemble, we took the crystal structure of the ADP-bound bacterial homologue HtpG (pdb id 2IOP) [35] and superimposed it with the crystal structure of a Cdc37 dimer bound with Hsp90-NTDs (pdb id 1US7) [50]. Using PyRosetta [95,96] the ADP-bound structure was minimized in the presence if Cdc37 dimer leading to partial opening of the cleft between the Hsp90-NTDs. The ensemble of conformational HtpG states was used in 3-body docking of Hsp90-Cdc37-Cdk4 complexes to partially mimic structural adaptability of the chaperone to binding with Cdc37 and Cdk4. Structural analysis of the docked solutions revealed a gradual improvement and consolidation of the Hsp90-Cdc37 and Cdc37-Cdk4 intermolecular interfaces as the HADDOCK-based score of the complexes improved (Figure 6). While less favorable docking solutions had visible packing defects, low-energy complexes closely reproduced the topology of the Hsp90-Cdc387-Cdk4 complex seen in the EM structure [53]. The favorable HADDOCK models are characterized by the asymmetric binding interface where Cdc37 binds to one of the Hsp90-NTD and the N-terminal of Cdk4 comes in proximity of another Hsp90-NTD (Figure 6).

**Figure 7:**
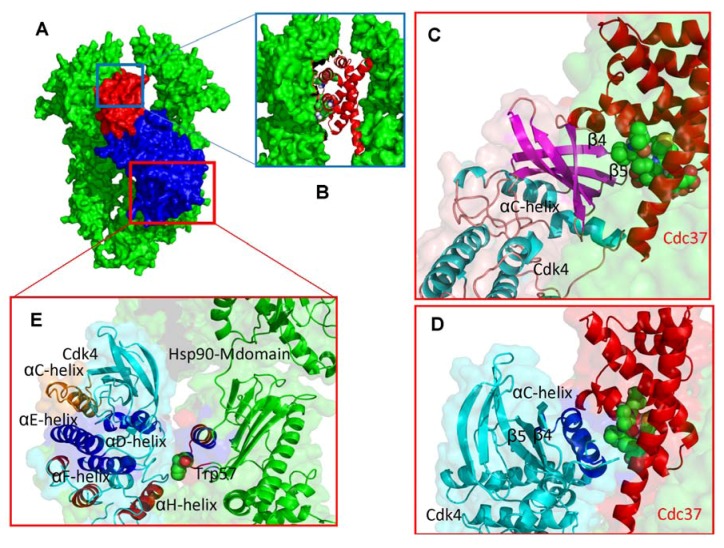
Structural Mapping of the Intermolecular Interfaces in the Hsp90-Cdc37-Cdk4 Docked Complexes (**A**) Structural overview of the low-energy docked complexes. The Hsp90 dimer is shown in green, Cdc37 is colored in red and Cdk4 is displayed in blue. (**B**) A close-up of the intermolecular interface between M-domain of Cdc37 (ribbon/sticks representation, colored in red) and Hsp90-NTD (surface representation, colored in green). (**C**) Structural details of the Cdk4-Cdc37 interface from the first low energy binding mode. The Cdc37 motif 190–LVIWCI-195 interfaces with the β4 and β5 strands of the N-terminal kinase lobe. Cdc37 is shown in red ribbons and the interacting Cdc37 helix is shown in spheres with atom-based coloring scheme. The interacting elements of Cdk4 are in blue ribbons, the rest of Cdk4 in cyan ribbons. (**D**) The Cdk4-Cdc37 interface from alternative low energy binding mode. The αC-helix region of Cdk4 interacts with the Cdc37 recognition motif. (**E**) A close-up of the interface between the C-terminal kinase lobe and the middle domain of Hsp90.

In agreement with the EM model, Cdk4 is attached to one monomer of Hsp90 with the C-terminal packed against the M-domain of Hsp90 and the N-terminal weakly interacting with the N-terminal of the same Hsp90 monomer (Figure 7A,B). Interestingly, two modes of Cdc37-Cdk4 interactions that are seen in the docked complexes may reflect the hierarchy of recognition events during kinase recruitment to the chaperone. Cdc37 is docked onto the N-terminal kinase lobe in the first binding mode and may represent the primary recognition event of the kinase fold by the chaperone (Figure 7C). In another low-energy solution, the αC-helix region becomes exposed to direct interactions with the Cdc37 recognition motif (Figure 7D). It is possible that these low-energy complexes exist in a dynamic equilibrium during recognition, loading and release of the kinase from the chaperone. In the primary binding mode, the recognition motif 190-LVIWCI-195 of Cdc37 is packed against the β4 and β5 strands that are connected to the structurally rigid αC-β4 loop (Figure 7C). The predicted Cdc37-Cdk4 interactions would necessarily interfere and perturb the network of secondary structure elements (αC-helix, β4-strand, β5-strand, the tip of the P-loop) that stabilizes the inactive kinase form.

Hence, this mode of Cdc37-Cdk4 recognition would be consistent with the mechanism of chaperone recruitment aiming to destabilize the unproductive inactive state and promote transition to the biologically active form. In agreement with the NMR study [52], the predicted Cdc37-Hsp90 interface is determined by hydrophobic interactions involving residues Met-164, Trp-193, Ala-204, and Leu-205 of Cdc37 (Figure 7B). Of particular importance is the mapping analysis of the Cdk4-Hsp90 interface since it allows comparing mechanistic roles of Hsp90 and Cdc37 in the kinase recruitment. The structural core of the C-terminal kinase lobe consists of two parts: the DEF-helical subdomain essential for catalysis and structural stability of the kinase core, and the GHI-subdomain that is often involved in substrate recognition and allosteric interactions [97,98]. We observed that the αH-helix (residues 219-EADQLGKIFDLI-230) may be engaged in direct interactions with the recognition loop 255-HDFNDP-260 and with the larger projecting loop 284-APWDMWNRDHKHG-296 of the Hsp90 M-domain (Figure 7E). These chaperone residues are often associated with protein binding, since their mutations can impair chaperone function without loss of ATPase activity, suggesting detrimental changes in the client interactions [33]. Importantly, the EM reconstruction study indicated that the larger C-terminal lobe of Cdk4 should interact with the M-domain of Hsp90 in the proposed binding site region near Trp-300 in yeast Hsp90 [53]. The recognition loops of Hsp90 and a critical Trp-57 (Trp-300 in yeast Hsp90) have been implicated in the Hsp90 interactions with protein clients [33,53]. The projecting loop of the Hsp90 M-domain and critical Trp-57 (Trp-300 in yeast Hsp90) residues were implicated in recognition of other protein kinase clients, for instance, they were found essential for mediating chaperone interactions with PKB in a yeast two-hybrid assay experiments [99]. Interestingly, the docked complexes depicted a previously proposed mechanism [33,53], in which the tip of a conformationally flexible projecting loop (centered around Trp-57) is inserted into the catalytic core, between the DEF and GHI helical subdomains of the C-terminal lobe (Figure 7E). The predicted interface is consistent with the notion that regulatory proteins can be often tethered to this region to induce allosteric binding. The results of docking simulations suggest that the kinase recognition and recruitment to the chaperone system may be primarily determined by Cdc37 targeting of the N-terminal kinase lobe. The interactions of Hsp90 with the C-terminal kinase lobe may provide additional “molecular brakes” that can lock (or unlock) kinase from the system during client loading (release) stages.

### 2.3. Towards Allosteric Inhibition of Protein Kinases by Targeting the Hsp90-Cdc37 Chaperone

Despite interesting insights about the details of the intermolecular interfaces, it is important to acknowledge and outline important conceptual limitations in structural modeling of chaperone-kinase complexes. Although conformational flexibility and adaptation of Hsp90 to binding partners is carefully incorporated in our approach using a template-based conformational ensemble of ADP-bound like states, the proposed scheme is a considerable simplification of the binding process. An accurate account of complexity and diversity of structural variations in Hsp90 in multi-protein functional assemblies continues to present a significant challenge for computational studies. Additionally, the N-terminal domain of Cdc37 (residues 1–147) is involved in recognition of kinase clients, yet this portion of Cdc37 is completely unresolved and was not considered in modeling studies. According to the EM reconstruction [53], the density for the Cdc37 N-terminus is buried inside the complex and may contact both Cdk4 and the Hsp90 N-terminal regions. It is tempting to speculate that structure-functional role of the N-terminal of Cdc37 may be similar to the “juxtamembrane latch” in the regulatory complexes of Egfr kinases that inserts between the N-terminal lobe of the receiver monomer and the C-terminal lobe of the activator monomer, allowing to strengthen the association between two kinase monomers and potentiate activation of the receiver molecule [100]. Structural modeling of highly dynamic Hsp90-Cdc37-kinase complexes remains to be challenging and uncertain for a number of reasons, owing to lack of high-resolution crystallographic information as the baseline for comparison. Recent pioneering studies by Agard and colleagues have underscored this fundamental challenge by showing that protein client binding may serve as a kinetic accelerator of large-scale conformational changes in the Hsp90 chaperone [101,102]. Nevertheless, structural mapping of the Hsp90-Cdc37-client interfaces by data-driven docking proved to be useful and represents a plausible approach for modeling transient complexes and interactions of the Hsp90-Cdc37 chaperone with kinase clients. The presented evidence of structurally convergent solutions to mediate kinase activation has reinforced the growing belief that Cdc37 is the main guardian of human kinome. Consistent with the emerging experimental data, our results indicate that Cdc37 may present a viable target for cancer because of its role in kinase recruitment to the Hsp90-Cdc37-kinase complex. There are now 13 Hsp90 inhibitors undergoing various phases of clinical evaluation, including among others 17-AAG (tanespimycin), and 17-DMAG (alvespimycin) [103]. Although there are currently no approved Hsp90-targeted drugs, there has been a growing effort and considerable progress in developing various therapeutic strategies such as targeting the formation of a Cdc37-kinase complex or blocking the Hsp90-binding site of Cdc37. Interrupting protein client binding with Cdc37 provides a targeted approach, in which the Hsp90 activity may be unaffected and specific client proteins prevented from binding to the Hsp90-Cdc37 complex. Chemical genomics and gene expression-based approaches have identified novel modulators of cancer phenotypes and classified a group of structurally related compounds comprising celastrol and gedunin as putative modulators of Hsp90 activity [104]. It has been also reported that celastrol disrupted Hsp90-Cdc37 interactions in the complex and exhibits antitumor activity *in vitro* and *in vivo* [57]. This study has suggested that celastrol is likely to interfere with Hsp90-Cdc37 interactions and binds to a region proximal to N-terminal ATP-binding site that overlaps with the binding site for Cdc37. The combinatorial signal transduction blockade by Hsp90 and Cdc37 inhibitors may help to overcome resistance of cancer cells to tyrosine kinase inhibitors which are believed to emerge from activation of parallel signal transduction pathways [105,106]. A prominent functional dependence of many tyrosine kinases on the chaperone system and structurally similar mechanisms of their recruitment to Cdc37 may be further explored in the design of allosteric kinase inhibitors targeting the Cdc37-kinase binding interfaces.

## 3. Conclusions

In this work, we used experimentally-guided protein docking to probe the allosteric nature of the Hsp90-Cdc37 binding with the cyclin-dependent kinase 4 (Cdk4) kinase clients. The results of docking simulations suggest that the kinase recognition and recruitment to the chaperone system may be primarily determined by Cdc37 targeting of the N-terminal kinase lobe. The interactions of Hsp90 with the C-terminal kinase lobe may provide additional “molecular brakes” that can lock (or unlock) kinase from the system during client loading (release) stages. The results of this study support a central role of the Cdc37 chaperone in recognition and recruitment of the kinase clients. Experimentally-guided structural and dynamic mapping of intermolecular interfaces and allosteric binding sites may help to quantify molecular mechanisms of allosteric inhibitors and determine the primary target for binding. Structural analysis may also have useful implications in developing strategies for allosteric inhibition of protein kinases by targeting the Hsp90-Cdc37 chaperone machinery.
